# Summary of the Transactions of the College of Physicians of Philadelphia, from November, 1845, to March, 1846, Inclusive

**Published:** 1846-06-01

**Authors:** 


*Summary of the Transactions of the College of Physicians of Philadelphia, from November,* 1845, *to March,* 1846, *inclusive.*

The College of Physicians of Philadelphia, is the title of an association for the promotion and diffusion of medical knowledge, and numbers among its members some of the most zealous and able of the profession in that ancient metropolis of medical science. Its stated meetings are held monthly, for reports upon some of the branches of medico-chirurgical science, voluntary communications, and the interchange of views upon the various subjects which are suggested. These constitute the "transactions," of which a summary is published quarterly. The transactions of the quarter preceding the last, were noticed in a former number of our journal, (January, 1846.) We have lately received a copy of the transactions for the last quarter, the title of which is prefixed to this article. This constitutes the eleventh number already published. If we may judge of their general character by the two last numbers, they must form a valuable repository of useful, practical information. We proceed to notice the several papers, and to make selections of such portions as we think will especially interest our readers.

*Annual Report on Surgery, by* Dr. Parrish.—Dr. P. takes up first, the question of the propriety of ovariotomy for the removal of large tumours from the cavity of the abdomen. He quotes the statistical table of the operations which have been performed, which was prepared by Prof. W. L. Atlee, and published in the Am. Jour. of Medical Sciences, for April, 1845. Prof. A. collected accounts of 101 operations. Of these, 20 were not completed, and in 6 no tumour was found. Throwing out of the total number the cases in which an operation was improper, 96 legitimate cases remain; and of this number, 64 recovered and 32 died. For farther details, we refer to the tabular view in the American Journal, or the transactions now under review.

Dr. P. brings together the arguments for and against the operation, and arrives at a conclusion unfavorable to its performance. The following quotation expresses his opinions and the reasons therefor:

"The fact of the cavity of the peritoneum being exposed to the air, and its contents roughly handled for the period of half an hour or more, without the occurrence of fatal peritonitis, which is adduced as an argument in favor of the peritoneal section, does not appear to the opponents of the operation to add any weight to this side of the question. That such exposure is dangerous, no one will doubt, and the tabular statement sufficiently proves it; and because patients escape, no argument is furnished for warranting the surgeon in exposing human life to such risks.

"Take, for instance, a female in the prime of life, and in the enjoyment of tolerable health, competent to attend to her domestic duties, and to mingle in the social circle—her functions regularly performed, and nothing to mar the pleasures of existence save an unsightly prominence of the abdomen, with occasional pain and mental distress, and the uneasiness attendant upon an ovarian tumour; and you have her in the condition which the advocates of this operation pronounce most favorable to its successful performance. Let an incision be made, eight inches long, through the external parietes of the abdomen and through the peritoneum, and after a tedious and painful dissection, let the tumour be dislodged and removed, and in every third case in which this process is instituted, let death be the consequence; what, we would ask, must be the feeling of the surgeon in these unfortunate cases? Will the escape of two patients render him easy under the loss of the third, when he reflects that the unfortunate individual, to whose relief his efforts were directed, might have lived in comparative comfort for a long period, and even through a long life? The occurrence of death, as an *immediate* consequence of a surgical operation, is an extremely painful event to the conscientious surgeon, even when the operation has been performed as a means of averting an inevitably fatal issue from disease; but where it has been instituted to rid an individual of an inconvenient or unsightly tumour, before life had been placed in jeopardy, and when all the functions were in full and vigorous exercise, a fatal issue must be doubly distressing. It is true, that from peculiar idiosyncrasies or some untoward accident, death may follow the simplest operation, but in the present case, the reflection is forced upon the mind that the cause was fully adequate to the effect. The wonder is, not that the patient should die, but that so many should survive. As has been well remarked by a highly respected Fellow of this College, (Dr. Meigs,) in a recent publication on this subject:

"'This is not merely a question of statistics; for, I am free to say, that I look upon operations for the extirpation of diseased ovaria as not to be justified by the most fortunate issue in any ratio whatever of the cases. I apprehend the ratio of success hitherto obtained, as not justifying the operation.'

"There is one other consideration which merits special attention in the consideration of this subject, and which, to our mind, furnishes a conclusive argument against ovariotomy. It is this:—In making the peritoneal section, the surgeon always proceeds upon an uncertainty. He is ignorant of the precise locality, the connections, and even of the nature of the tumour which it is his object to remove. He lays open one of the most important cavities in the body, exposes vital and highly sensitive parts, and inflicts upon his patient severe pain, on an exploring expedition, which may result in death, or, at least, in a failure to afford relief without the loss of life.

"By reference to the table above referred to, it will be perceived that out of the 101 operations, 20 were not completed, and in 6 no tumour was found! Thus, in one-fourth of the cases, it is fair to infer the surgeon made a mistake in diagnosis; of these 20, 8 died, and the other 12 encountered a severe and highly dangerous operation without receiving benefit. Of the 6 cases in which *no tumour was found*, 2 died—victims, most certainly, to ignorance and rashness.

"Nor can these errors in diagnosis be wholly avoided by the utmost skill and penetration on the part of the surgeon. The existence or non-existence of adhesions cannot be foretold, although such knowledge is of the utmost importance in determing the possibility of removing the tumour; the parts are hidden from view, and there is no method of ascertaining their condition but by inspection. Even our friends, the Drs. Atlee, of

Lancaster—whose discrimination and skill will not be questioned by those who know them—have been several times deceived as to the nature of the cases which have been subjected to their treatment."

If we were to express any sentiments of our own on this important subject, we should say that the probabilities of success, as determined by statistics, are sufficient to justify the surgeon in the performance of the operation; but that the circumstances which generally call for it require that he should explain the matter candidly and freely to the patient and friends, and in the great majority of cases, advise against it. If, after a full understanding of the subject, the patient and friends continue to desire an operation, he is at liberty to proceed with it. We do not say that in our opinion there are no cases in which the surgeon should not exert an influence upon the patient in behalf of the operation, but we believe cases of this description are few, compared with the number of those in which the judgment against it should be entertained and expressed.

Dr. P. treats at considerable length of the treatment of Aneurism by pressure, as recommended and practised by Dr. Bellingham, of Dublin. We have given an abstract of the peculiarities of this method in a former number (Vol. 1. No. 7,) of our journal. Dr. Parish refers to an interesting paper on the subject, with the details of five cases, reported for the Am. Jour. of Med. Sciences, Feb. 1839, by Dr. T. S. Kirkbridge.

On the subject of *Amputation in consequence of gun-shot wounds*, the reporter gives the views of the distinguished French surgeon Lisfranc. Lisfranc does not consider amputation called for when an articulation of the first or second importance is laid open by a projectile, nor in comminuted fracture of a long bone of the upper or inferior extremity. He believes that many limbs may be saved after gun-shot fractures of the thigh, when amputations are performed after the recommendation of Larrey, Guthrie, and other army surgeons. He, also, doubts the propriety of amputating when wounds are complicated with laceration of the principal blood vessels of the limb. Dr. Parrish adds that these views are in accordance with the highest surgical authority of our own country. We quote the concluding paragraph of the report, which expresses a sentiment in which all who take a high and humane view of the progress of medical science must heartily concur.

"May we not indulge the hope, that as our knowledge of the principles which control or modify the progress of these serious accidents, comes to be more fully matured, that the number of cases in which amputation will be deemed necessary will be narrowed down to a comparatively small circle, and that by the exercise of skill in addressing remedies to the general system, and in taking advantage of all the means and appliances within the reach of the art, that surgery will yet triumph over many of those injuries which have been so destructive, as to demand an immediate resort to the knife?"

